# Multi-omics-based phenotyping of AFG3L2-mutant lymphoblasts determines key factors of a pathophysiological interplay between mitochondrial vulnerability and neurodegeneration in spastic ataxia type 5

**DOI:** 10.3389/fnmol.2025.1548255

**Published:** 2025-02-20

**Authors:** Menekse Oeztuerk, Diran Herebian, Kale Dipali, Andreas Hentschel, Nina Rademacher, Florian Kraft, Rita Horvath, Felix Distelmaier, Sven G. Meuth, Tobias Ruck, Ulrike Schara-Schmidt, Andreas Roos

**Affiliations:** ^1^Department of Neurology, Medical Faculty and University Hospital Düsseldorf, Heinrich Heine University, Düsseldorf, Germany; ^2^Department of Neurology, BG-University Hospital Bergmannsheil, Ruhr University Bochum, Bochum, Germany; ^3^Heimer Institute for Muscle Research, BG-University Hospital Bergmannsheil, Bochum, Germany; ^4^Department of General Pediatrics, Neonatology and Pediatric Cardiology, Medical Faculty and University Hospital Düsseldorf, Heinrich Heine University, Düsseldorf, Germany; ^5^Leibniz-Institut für Analytische Wissenschaften -ISAS- e.V., Dortmund, Germany; ^6^Department of Pediatric Neurology, Centre for Neuromuscular Disorders, Centre for Translational Neuro- and Behavioral Sciences, University Duisburg-Essen, Essen, Germany; ^7^Institute for Human Genetics and Genomic Medicine, Medical Faculty, Rheinisch-Westfälische Technische Hochschule Aachen University Hospital, Aachen, Germany; ^8^Department of Clinical Neurosciences, University of Cambridge, Cambridge, United Kingdom; ^9^Brain and Mind Research Institute, Children’s Hospital of Eastern Ontario Research Institute, Ottawa, ON, Canada

**Keywords:** AFG3 like matrix AAA peptidase subunit 2, SPAX5, MCU, multi-omics lymphoblasts, liquid biopsy

## Abstract

Mitochondrial integrity is fundamental to cellular function, upheld by a network of proteases that regulate proteostasis and mitochondrial dynamics. Among these proteases, AFG3L2 is critical due to its roles in maintaining mitochondrial homeostasis, regulating mitochondrial protein quality, and facilitating mitochondrial biogenesis. Mutations in AFG3L2 are implicated in a spectrum of diseases, including spinocerebellar ataxia type 28 (SCA28) and spastic ataxia 5 (SPAX5), as well as other systemic conditions. This study employs a multi-omics approach to investigate the biochemical impact of AFG3L2 mutations in immortalized lymphoblastoid cell lines derived from a patient with biallelic variants leading to spastic ataxia (SPAX5). Our proteomic analysis revealed AFG3L2 impairment, with significant dysregulation of proteins critical for mitochondrial function, cytoskeletal integrity, and cellular metabolism. Specifically, disruptions were observed in mitochondrial dynamics and calcium homeostasis, alongside downregulation of key proteins like COX11, a copper chaperone for complex IV assembly, and NFU1, an iron-sulfur cluster protein linked to spastic paraparesis and infection-related worsening. Lipidomic analysis highlighted substantial alterations in lipid composition, with significant decreases in sphingomyelins, phosphatidylethanolamine, and phosphatidylcholine, reflecting disruptions in lipid metabolism and membrane integrity. Metabolomic profiling did not reveal any significant findings. Our comprehensive investigation into loss of functional AFG3L2 elucidates a pathophysiology extending beyond mitochondrial proteostasis, implicating a wide array of cellular processes. The findings reveal substantial cellular disturbances at multiple levels, contributing to neurodegeneration through disrupted mitochondrial respiratory chain, calcium homeostasis, cytoskeletal integrity, and altered lipid homeostasis. This study underscores the complexity of SPAX5 pathophysiology and the importance of multi-omics approaches in developing effective strategies to address the impact of loss of functional AFG3L2. Our data also highlight the value of immortalized lymphoblastoid cells as a tool for pre-clinical testing and research, offering a detailed biochemical fingerprint that enhances our understanding of SPAX5 and identifies potential areas for further investigation.

## Graphical Abstract

Pathophysiological impact of *AFG3L2* mutations in SPAX5 as revealed by multi-omics analysis. The mitochondrial protease AFG3L2 is localized in the inner mitochondrial membrane (IMM), where it plays a critical role in maintaining mitochondrial proteostasis. Its physiological functions include ATP-dependent protein hydrolysis, calcium homeostasis, and the degradation of misfolded proteins, which are essential for mitochondrial integrity, axonal development, and neuronal health. Mutations in *AFG3L2* impair these functions, leading to dysregulation of neuronal function-related proteins, cytoskeletal instability, and respiratory dysfunction. These molecular disruptions result in neurodegenerative phenotypes, including SPAX5 (spastic ataxia 5), SCA28 (spinocerebellar ataxia 28), and OPA12 (optic atrophy 12). On the right, findings from a patient with SPAX5 are summarized. Multi-omics analysis of lymphoblastoid cells revealed mitochondrial calcium homeostasis disruption, impaired lipid metabolism, and immune pathway activation. These cellular disturbances highlight a systemic impact of AFG3L2 dysfunction, contributing to the neurodegenerative features observed in SPAX5. Collectively, these findings underline the critical role of AFG3L2 in cellular homeostasis and emphasize the value of multi-omics approaches in understanding the pathophysiology of SPAX5. Created using BioRender.com. 
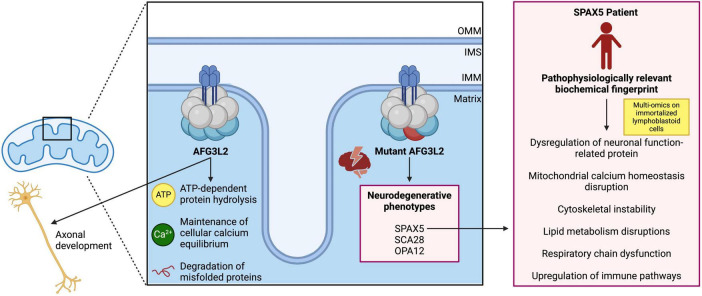

## 1 Introduction

Mitochondrial integrity is a cornerstone of cellular viability, playing a pivotal role in ATP generation and intracellular signaling pathways. Complex quality control mechanisms meticulously oversee mitochondrial health, orchestrating the selective elimination of damaged components. Within this surveillance matrix, mitochondrial proteases emerge as key regulators, ensuring proteome integrity and modulating mitochondrial dynamics, crucial for cellular function. Recent investigations have broadened our understanding on how mutations in mitochondrial protease genes affect the cellular homeostasis. Initially perceived as disruptors of proteostasis, these mutations are now recognized for their contribution to pathologies, often implicated in neurodegeneration, through their role in altering mitochondrial function and disruption of signal transduction (Jagtap et al., 2023; Shpilka and Haynes, 2018). Thus, the dependency of neurons on these mechanisms, with failures leading to neurodegenerative diseases, emphasizes the significant role of the mitochondrial quality control network in neuronal health and disease (Rugarli and Langer, 2012). The mitochondrial proteases regulate essential processes, including mitochondrial gene expression and respiratory chain function, further associating protease dysfunction with various pathological states, such as metabolic syndromes, cancer and neurodegenerative disorders (Beck et al., 2016; Feng et al., 2021; Todosenko et al., 2023).

*AFG3L2* (AFG3 like matrix AAA peptidase subunit 2), a gene encoding AFG3-like protein 2, is a crucial component of human mitochondrial ATPases (m-AAA) responsible for maintaining mitochondrial homeostasis through quality control and regulatory functions within the inner mitochondrial membrane (Quirós et al., 2015; Richter et al., 2015). The m-AAA proteases assemble into hexameric ATP-dependent proteolytic complexes within the inner mitochondrial membrane, positioning their catalytic sites toward the matrix space, showing profound involvement in mitochondrial dynamics (Arlt et al., 1996).

There are two variants of human m-AAA proteases, distinguished by their subunit configuration: one is homo-oligomeric, while the other forms hetero-oligomeric complexes by the interaction of AFG3L2 with SPG7 (paraplegin) homologous subunits (Atorino et al., 2003; Banfi et al., 1999; Koppen et al., 2007). AFG3L2 is critically involved in axonal development. Its deficiency is known to result in reduced myelinated fibers, suggesting impaired axonal caliber regulation and leading to inefficient axonal-glial interactions (Maltecca et al., 2008).

Mutations in *AFG3L2* may lead to a clinical spectrum: dominant variants are associated with late onset spinocerebellar ataxia type 28 (SCA28; MIM: 610246), whereas pathogenic biallelic variants lead to spastic ataxia 5 (SPAX5; MIM: #614487) (Franchino et al., 2023; Tulli et al., 2019). Moreover, mutations in AFG3L2 may also cause autosomal dominant optic atrophy (MIM: 618977) (Baderna et al., 2020).

Functional studies in *Drosophila melanogaster* showed that *Afg3l2* loss leads to significantly reduced respiratory chain activity and extensive abnormalities in mitochondrial gene expression in turn resulting in pronounced neurodegeneration characterized by shortened lifespan, locomotor impairment, and accumulation of vacuoles in brain tissue. These neurodegenerative phenotypes were associated with severely diminished mitochondrial transcription and translation due to defective ribosome biogenesis (Pareek and Pallanck, 2020).

Emerging evidence highlights the significance of *AFG3L2* function, linking pathogenic variants to a broader spectrum of pathologies that underscore the critical role of neurodegeneration based on pathomechanisms beyond mitochondrial malfunction such as altered calcium homeostasis, and cytoskeletal dysfunction in mitochondrial dynamics and neuronal health (Kondadi et al., 2014; Maltecca et al., 2012).

Studies on knockout mouse models demonstrate that loss of functional *AFG3L2* leads to early mitochondrial fragmentation and misplacement within the dendritic trees of Purkinje cells, marking the onset of neurodegeneration. These mitochondrial disturbances result in reduced protein synthesis and perturbed ribosomal assembly within mitochondria, essential for neuronal function (Ghosh Dastidar et al., 2023). Beyond neurons, loss of functional *AFG3L2* affects astrocytes, thus contributing to motor dysfunctions and severe neurological disorders (Tosato and Cohen, 2007). Furthermore, impaired mitochondrial function associated with *AFG3L2* deficiency leads to increased oxidative stress and inflammation, exacerbating neuroinflammatory conditions (Ghosh Dastidar et al., 2023; Maltecca and Casari, 2010).

This study investigates immortalized lymphoblastoid cell lines as a robust *in vitro* disease model, enabling to decipher biochemical fingerprints relevant to neuronal pathophysiology. Employing a multi-omics approach, we integrate proteomics, lipidomics and targeted metabolomics analyses to unravel the extensive molecular alterations induced by bi-allelic *AFG3L2* variants. Thus, through comprehensive molecular phenotyping of *AFG3L2*-mutant lymphoblasts, our research delineates crucial interactions between mitochondrial dysfunction and its downstream effects on cellular architecture and survival mechanisms.

## 2 Materials and methods

### 2.1 Ethical considerations

This study has been approved by the ethical committee of the University Medicine Essen (19-9011-BO). Written consent has been obtained by the caregivers of the SPAX5 patient.

### 2.2 Generation of a lymphoblastoid cell line

The establishment of lymphoblastoid cell lines was initiated from peripheral blood specimens obtained from the index patient, following previously established protocols (Tosato and Cohen, 2007). Cells were cultivated in RPMI media supplemented with 10% FCS and Pen/Strep. As controls lymphoblasts from three different healthy female donors were immortalized and included into the study.

### 2.3 Proteomic profiling

Proteomic profiling was conducted on immortalized lymphoblastoid cells derived from a SPAX5 patient. Lymphoblastoid cells have been a suitable *in vitro* model to study biochemical processes underlying rare neurological diseases (Kollipara et al., 2017) including such affecting the mitochondrial calcium uniporter (MCU) complex (Kohlschmidt et al., 2021). Before proteomic profiling was conducted, we checked *AFG3L2* abundance in our in-house white blood cells peptide library to warrant suitability of this *in vitro* model to study biochemical processes taking place upon loss of functional *AFG3L2*. The mass spectrometry proteomics data have been deposited to the ProteomeXchange Consortium via the PRIDE (Perez-Riverol et al., 2022) partner repository with the dataset identifier PXD056395.

For accurate label-free quantification, proteins identified by a minimum of ≥ 2 unique peptides were included in the analysis. The normalized abundances for each protein, calculated using Spectronaut, were averaged and used to determine the abundance ratios between patient cells and control samples. A Student’s *t*-test was conducted in MS Excel to calculate *p*-values, with a threshold for significance set at ≤ 0.05. Proteins with *p*-values of ≤ 0.05 and abundance ratios of ≥ 2 or ≤ 0.5 (indicating up-regulation or down-regulation, respectively) were classified as significantly regulated. Statistical analysis was performed using GraphPad Prism 9.2 (GraphPad Software, Inc., San Diego, CA, USA). Figures were edited using Inkscape (version 1.2).

Statistical analysis involved the use of false discovery rate (FDR) adjustments via Q-values to rigorously control for the multiplicity of tests, ensuring that only proteins with statistically robust changes were considered. Each protein’s fold change was calculated to quantify the expression differences, with emphasis placed on those exhibiting significant deviations from control norms.

After statistical validation, we delved deeper into the biological significance of these proteins by integrating data from multiple proteomic databases, including UniProt for functional annotations and STRING for protein-protein interactions. This allowed us to map the proteins onto existing biological pathways and to identify their roles in disease-relevant processes using pathway enrichment analyses provided by Reactome and KEGG (Kyoto Encyclopedia of Genes and Genomes). To further refine our list, proteins involved in mitochondrial dynamics, neurodegenerative processes, and cytoskeletal organization — key areas implicated in SPAX5 pathology — were prioritized.

### 2.4 Targeted metabolic profiling

Examining the metabolome of lymphoblastoid cells from one SPAX5 patient exposes a spectrum of metabolic alterations, enriching our understanding of the disease’s metabolic phenotype. To this end, extraction of the targeted metabolites was performed by adding 300 μL Methanol/H_2_O (80/20: v/v) to the cells with known protein concentrations, which were homogenized by ceramic beads CK14 using Precellys^®^ lysing kit. The corresponding isotopically labeled internal standards were added to the extraction solution. The targeted compounds were analyzed by UPLC-MS/MS. The system consists of a UPLC I-Class (Waters) coupled to a tandem mass spectrometer Xevo-TQ-XS (Waters). Electrospray ionization (ESI) was performed in the negative ionization mode for the compounds fructose-1,6-bisphosphate, phosphoenolpyruvate (PEP) and 2/3-phosphoglycerate (Campos et al., 2017). The tricarboxylic acid (TCA), energy metabolites (AMP, ADP and ATP) as well acetyl- and succinyl-CoA were measured in the positive ionization mode as referred in the literature (Jones et al., 2021; Law et al., 2022; Marquis et al., 2017). Amino acids were measured using the HILIC column by LC-MS/MS in positive ion mode (Németh et al., 2023). Mass spectrometric quantitation of the compounds was carried out in the multiple reaction monitoring (MRM) mode. MassLynx software (v4.2; Waters, UK) was used for instrument’s control and data acquisition. Quantitation analysis was performed by TagetLynx XS software (Waters, UK). All graphical analyses were performed with GraphPad Prism 9.2 (GraphPad Software, Inc., San Diego, CA, USA) and with the software Inkscape (version 1.2).

### 2.5 Lipidomic profiling

To comprehensively assess lipid changes in SPAX5, we employed a detailed protocol combining lipid extraction, chromatographic separation, and high-resolution mass spectrometric analysis. For that purpose, cultured cells were harvested in 1.5 ml Eppendorf tubes and cell lipid content was then extracted using a Matyash protocol (Matyash et al., 2008). In brief, to the cell pellets, 187.4 μL of MeOH (containing internal standard; IS) was added, and vortexed briefly. Then, 625 μL of MTBE was added, followed by incubation in an Eppendorf shaker (Thermomixer comfort) for 1 h at 25°C, at 1,400 RPM. Sequentially, 156.2 μL of H_2_O was added, and the mixture was incubated for 10 min at 25°C, to induce phase separation. The samples were then centrifuged for 10 min at 1,500 *g* at RT to separate two liquid phases. Next, the organic (upper) phase (∼700 μL) was collected in EPs and dried under a vacuum centrifuge at RT. To speed up drying, 200 μL MS grade methanol was added after 25 min of centrifugation. The extracted lipids were re-dissolved 50 μL CHCl_3_/MeOH/H_2_O (60:30:4.5) and analyzed by liquid chromatography (LC) high-resolution mass spectrometer (HRMS).

Untargeted lipidomics of cellular lipid extracts was performed on a Vanquish DuO system coupled to a Exploris 240 MS. Lipids were separated on a CSH C18 column (1.7 μm, 2.1 × 100 mm). Mobile phase A consisted of acetonitrile: water (60:40) with 10 mM ammonium formate and mobile phase B consisted of acetonitrile: isopropyl alcohol (10:90) with 10 mM ammonium formate. The following gradient program was applied: 15% B for 1 min after injection, then increase to 60% B in 9 min, then to 75% B in 8 min and then to 100% B in further 2.5 min. An isocratic 100% B step was then maintained for 2.5 min, and the column was subsequently reconditioned to 15% B for 2 min. Total run time was 25 min with the following conditions: flow rate, 0.4 ml/min; column temperature, 55°C; injection volume, 6 μl.

The instrument was operated in positive ESI and negative ESI mode. MS source parameters were as follows: capillary voltage was set at 3.5 kV and 2.5 kV for positive ion and negative ion mode, respectively. Ion transfer tube and vaporizer temperature were set at 300 and 350°C, respectively. Mass spectra were recorded in Full scan mode with data dependent MS/MS fragmentation with normalized collision energy 25%, isolation window = 1 m/z, and mass resolution of 15,000. Full scan spectra were acquired in the range of m/z of 200–1,600 m/z, with mass resolution of 120,000 in positive ion mode and 60,000 in negative ion mode. Auxiliary gas and sweep gas flows were 7 and 1 arb units, respectively and RF lens was 70%.

Lipid identification and quantification was performed by Progenesis QI Software (version 2.4, Waters). The process in Progenesis QI involved the following steps: create a new experiment, import data, alignment of runs, peak picking (sensitivity automatic, adducts: [M+H]+, [M+Na]+, [M+K]+, and [M+NH_4_]+), experimental design set up, review deconvolution, identify compounds (precursor tolerance: 10 ppm and fragment tolerance: 20 ppm), and review compounds. For compound identification, theoretical MS/MS fragment ions of lipids were matched with experimental runs. The total ion chromatogram (TIC) normalized abundances of each sample, and compound identifications from Progenesis QI were exported to Excel. Relative peak area ratios of lipid species to IS were converted to molar concentrations using a known IS concentration. Statistical analysis of datasets was carried out using Microsoft Excel Version 16.86 (24060916). All graphs were generated using R 3.5.3 (R Foundation for Statistical Computing, Vienna, Austria) and the software Inkscape (version 1.2).

## 3 Results

### 3.1 Clinical presentation and genetic testing of the index patient

The patient is a 4-year-old female born to non-consanguineous parents and diagnosed with autosomal recessive SPAX5 due to compound heterozygous variants in the *AFG3L2* gene (detailed below). Family history is non-contributory for neurological or genetic disorders, apart from the father’s diagnosis of type II diabetes mellitus.

**FIGURE 1:**
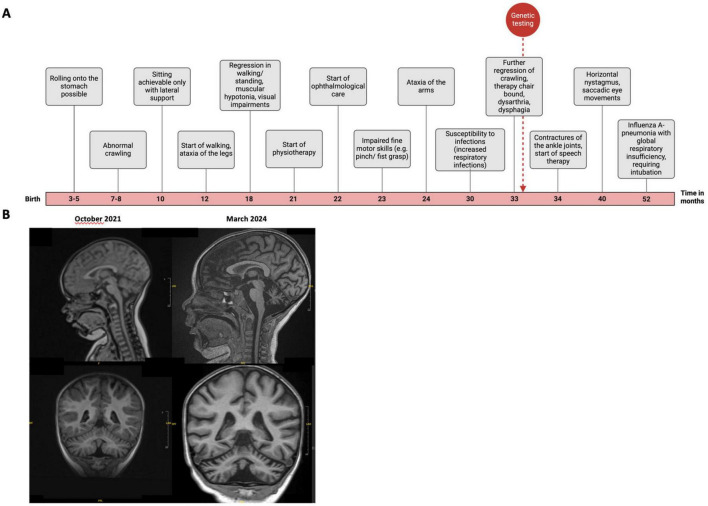
Clinical data of our SPAX5-patient carrying compound heterozygous *AFG3L2* variants. **(A)** Schematic timeline representation of the clinical findings and diagnostic workup. **(B)** T1-weighted cranial MRI scans acquired in sagittal (upper half) and coronal (lower half) plane. The left panels display scans from October 2021, while the right panels show scans from March 2024. There is notable progression of generalized brain volume reduction, with a pronounced atrophy observed in the cerebellum. Created using BioRender.com.

The pregnancy was largely unremarkable except for a noted reduction in fetal movements. Labor was induced, culminating in a spontaneous vaginal delivery at 41+6 weeks of gestation. Figure 1A provides an overview of the patient’s developmental progression. In early infancy, the patient exhibited weak sucking behavior. Motor milestones were achieved as follows: between 3 and 5 months, she attained the ability to turn from the prone to the supine position and vice versa; at 7 to 8 months, she began crawling; and by 10 months, she could sit unsupported with a rounded back. By 12 months, she was able to take lateral steps while holding onto objects. However, a progressive decline in motor function was subsequently observed. At approximately 18 months, the patient began to lose previously acquired milestones, including the ability to pull up to a standing position and to walk, ultimately resulting in an inability to stand or ambulate independently. Ataxia of the lower extremities was observed at 12 months, while ataxia of the upper extremities manifested at 24 months. Additionally, a chewing weakness was evident in infancy, with progressive difficulties in swallowing, resulting in frequent choking episodes during meals and drinking.

At the age of 3 years and 3 months, neurological examination identified a constellation of deficits, including ataxia of both the arms and legs, dysarthria, dysphagia, generalized muscular hypotonia, and truncal ataxia, leading to a kyphotic posture and instability while sitting. Further clinical signs included ankle joint contractures, generalized microcephaly, horizontal nystagmus, saccadic eye movements, mild ptosis, and gait impairment. Moreover, from the age of two years, the patient exhibited an increased susceptibility to infections, predominantly affecting the respiratory tract. At four years of age, she developed Influenza A pneumonia, resulting in global respiratory insufficiency that necessitated intubation. A specialized ophthalmologic examination revealed a yellowish discoloration of the optic nerves. Further ophthalmologic evaluations are warranted to clarify whether this finding may indicate early signs of optic atrophy and to assess the potential progression of optic nerve involvement.

Additional evaluations revealed a subaortic ventricular septal defect. Nerve conduction studies demonstrated evidence of demyelinating peripheral neuropathy, with bilaterally reduced nerve conduction velocities in the peroneal motor nerve (37 m/s on the left, 41 m/s on the right; normal age-adjusted reference range: 43–51 m/s) and the tibial motor nerve (33 m/s on the left, 31 m/s on the right; normal age-adjusted reference range: 41–45 m/s). Magnetic resonance imaging (MRI) of the brain revealed progressive cerebellar atrophy as well as atrophy of the medulla oblongata (Figure 1B).

Chromosome analysis and comparative genomic hybridization (CGH) array analysis revealed a normal karyotype (46, XX). Testing for 5q-related spinal muscular atrophy was negative. Next, trio exome sequencing was initiated and unveiled two compound heterozygous variants affecting the *AFG3L2* gene: c.1284G > T [p.Glu428Asp; paternally inherited and classified as VUS = variant of unknown significance (PM1, PM2)] and c.2201_2203del [p.Glu734del; maternally inherited and also classified as VUS (PM1, PM2)]. Although both variants are classified as VUS, based on the different clinical aspects of the phenotype of our patient fitting with SPAX5 (see Table 1), we consider these two variants as being likely pathogenic and thus causative for the neurodegenerative disorder. Results of *in silico* testing of the missense variant are presented in Table 2.

**TABLE 1:** Comparison of clinical features observed in our patient with phenotypic hallmarks listed in OMIM for SPAX5.

Clinical hallmarks	Present in patient (yes/no)
Early-onset spasticity (Pierson et al., 2011)	Yes
Impaired ambulation (Pierson et al., 2011)	Yes
Cerebellar ataxia (Pierson et al., 2011)	Yes
Oculomotor apraxia (Pierson et al., 2011)	Yes
Dystonia (Pierson et al., 2011)	No
Myoclonic epilepsy (Pierson et al., 2011)	No
Severe progressive myoclonus (Muona et al., 2015)	No
Optic atrophy (Caporali et al., 2020)	No (but discoloration)

Clinical presentation of the patient to the documented phenotypic characteristics associated with SPAX5 (MIM: #614487) described in OMIM (Online Mendelian Inheritance in Man) revealed presence of four clinical key features.

**TABLE 2:** Pathogenicity prediction of the c.1284G > T (p.Glu428Asp) variant.

Prediction methode	Score
AlphaMissense	0.9370*
BayesDel	0.1163*
CADD	21.7*
FATHMM	−3.18*
Gerp++	2.39*
PolyPhen2	0.244
PhyloP-100	0.622
PrimateAI	0.8962*
SIFT	0.014*
REVEL	0.585*

The *in silico* prediction using several pathogenicity prediction algorithms indicate a possible deleterious effect for the c.1284G > T (p.Glu428Asp) variant in *AFG3L2*.

*Indicate scores which predict a deleterious effect using the thresholds from the original publications.

### 3.2 Proteomic profiling reveals significantly dysregulated proteins in SPAX5 lymphoblastoid cells

**FIGURE 2:**
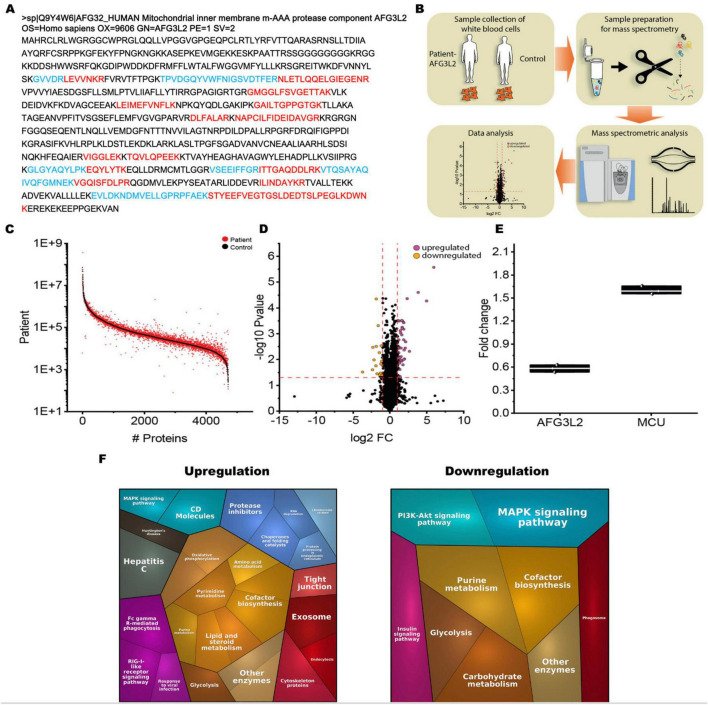
Results of proteomic profiling on *AFG3L2*-mutant immortalized lymphoblastoid cells. **(A)** Coverage of the human AFG3L2 amino acid sequence by 21 unique tryptic peptides of our spectral library applied for mass spectrometric based protein quantification in a data-independent-acquisition (DIA) mode. Red and blue color labeling was chosen to ensure appropriate visualization of adjacent unique tryptic peptides **(B)** Schematic representation of the applied workflow. **(C)** Abundance plot showing the dynamic range of all identified proteins based on their relative quantification using always the 3 highest abundant peptides for each protein, allowing protein comparison within an experiment. All identified proteins of the control (black) are sorted with decreasing abundance while the patient (red) was plotted in the same order to directly compare the different abundances. All identified proteins cover a dynamic range of almost nine orders of magnitude. **(D)** Volcano plot highlighting the statistically significant dysregulation of 63 proteins (DAPs = differentially abundant proteins). Of those, 47 are increased and 16 are decreased. The *x*-axis represents the log2 Fold Change in protein expression levels, while the *y*-axis shows the -log10 *P*-value, indicating the statistical significance of the changes. Purple dots represent upregulated proteins with log2 Fold Change greater than 1 and *P*-value less than 0.05. Orange dots represent downregulated proteins with log2 Fold Change less than –1 and *P*-value less than 0.05. Black dots represent proteins with log2 Fold Change between –1 and 1 or *P*-value greater than or equal to 0.05. The horizontal dashed red line marks the significance threshold at *P*-value = 0.05 [-log10(0.05)], and the vertical dashed red lines indicate the fold change thresholds at log2 Fold Change = –1 and 1. **(E)** Proteomic analysis revealed a 60% residual expression of AFG3L2 in SPAX5 patient-derived cells compared to wild-type, reflecting likely expression of the missense variant protein. The pathogenic nature of this variant is underscored by global proteomic alterations and specific dysregulation of its key binding partner, MCU. **(F)** Proteomaps representing the functional categorization of the differentially abundant proteins (upregulated proteins on the left side, downregulated proteins on the right side).

Inspection of our in-house library of tryptic peptides from human white blood cells revealed a coverage of *AFG3L2* by 21 unique peptides (Figure 2A) declaring these cells suitable for biochemical profiling.

Mass spectrometry-based quantification of protein abundances in patient derived immortalized lymphoblastoid cells compared to three matched healthy controls (Figure 2B) covering a dynamic range of almost nine orders of magnitude (Figure 2C) unveiled the statistically significant dysregulation of 63 proteins (DAPs = differentially abundant proteins). Of those, 47 are increased and 16 are decreased (Figure 2D and Supplementary Table 1). A GO term-based analysis of these dysregulated proteins further identified specific molecular processes and subcellular structures impacted by increased and decreased protein abundances (Supplementary Figure 1). Although the overall number of altered mitochondrial proteins was not high, we detected six increased (CMPK2, COX11, COX15, GATM, NFU1, and TGM2) and two decreased proteins (MRS2 and RDH13), which are of known mitochondrial origin (Supplementary Table 1). Furthermore, among the identified dysregulated non-mitochondrial proteins, we detected PRKCB (Protein Kinase C Beta Type). PRKCB is involved in the regulation of mitochondrial calcium uptake by modulating the activity of calcium channels and transporters within the mitochondria (Patergnani et al., 2013). This molecular observation fits with the dysregulation of MCU as known AFG3L2 binding partner (see above).

Filtering the proteomic data for *AFG3L2* abundance revealed a residual expression of 60% in cells derived from the SPAX5 patient compared to wild-type cells. This reduction most likely reflects expression of the missense variant protein, whose pathogenic nature is in turn suggested not only by the overall proteomic alterations but also by the concurrent dysregulation of its main binding partner, MCU, as shown in Figure 2E.

Proteomaps-based *in silico* studies of dysregulated proteins (Figure 2F) highlight significant alterations across various biological processes: Upregulated proteins profoundly affect lipid and steroid metabolism. In addition, proteins with increased abundance influence cofactor biosynthesis and amino acid metabolism, reflecting a broad metabolic response upon loss of functional *AFG3L2*. This finding highlights an impact on pyrimidine metabolism and oxidative phosphorylation, indicating the relevance of altered mitochondrial function. Furthermore, the glycolysis pathway is notably active, indicating a shift toward anaerobic energy production and pathways and changes in protease inhibitors suggest increased immune-related activities.

Further, we found alterations of immune-related pathways, such as Fc gamma R-mediated phagocytosis and the RIG-I-like receptor signaling pathway. Chaperones and folding catalysts, along with protein processing in the Endoplasmic Reticulum, show upregulation, indicating an enhanced protein quality control mechanism upon loss of function *AFG3L2*. Additionally, tight junction, endocytosis and exosome pathways are upregulated, reflecting changes in cell communication. Increase of cytoskeletal components might reflect structural changes. However, decreased proteins also profoundly impact on cell metabolism as decreased proteins affect purine metabolism and carbohydrate metabolism as well as glycolysis. Further biological processes covered by decreased proteins include the MAPK signaling pathway, the PI3K-Akt signaling pathway, both crucial for cell survival and growth in addition to the insulin signaling pathway. Reduced synthesis of essential coenzymes is suggested by affection of proteins crucial for the respective biosynthesis. Moreover, phagocytosis may be affected, supported by decreased protein abundances.

In summary, the significant dysregulation of 63 proteins in lymphoblastoid cells underscores critical cellular vulnerabilities associated with loss of proper *AFG3L2* function including mitochondrial, metabolic and immune processes.

### 3.3 Targeted metabolic profiling of SPAX5 lymphoblastoid cells does not indicate significant metabolic changes

**FIGURE 3:**
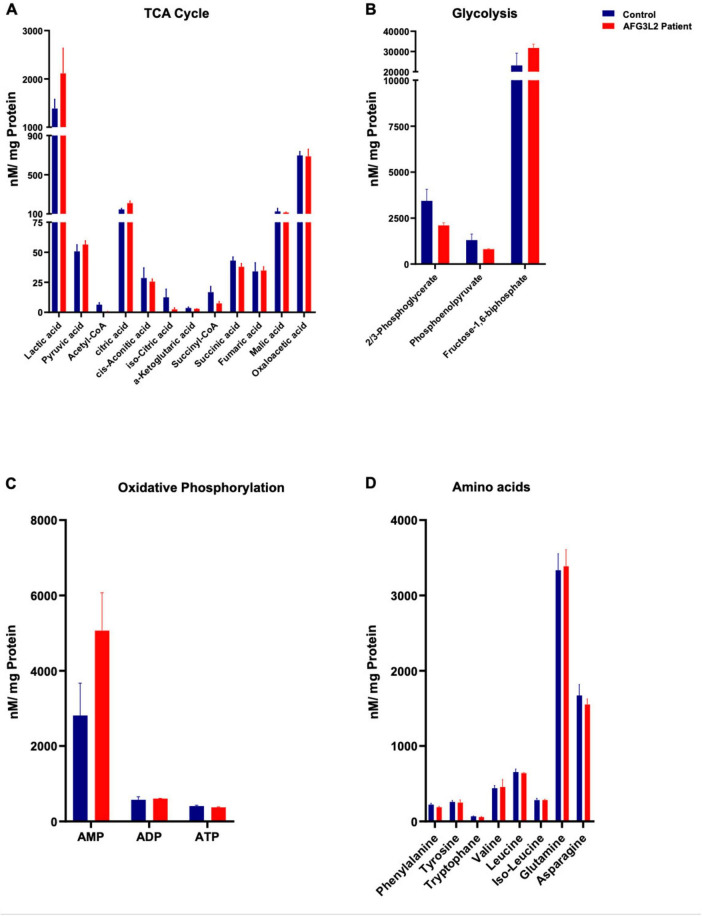
Results of targeted metabolic profiling on *AFG3L2*-mutant immortalized lymphoblastoid cell. The figure illustrates metabolic profiling results for immortalized lymphoblastoid cells from a SPAX5 patient (red) compared to controls (blue). Panel **(A)** depicts the TCA cycle, while panel **(B)** focuses on glycolysis. Panel **(C)** highlights oxidative phosphorylation, and panel **(D)** examines amino acid concentrations in nM/mg Protein.

Following indications of disrupted metabolic homeostasis from our proteomic analysis, we conducted a detailed metabolic profiling to further explore potential biochemical alterations in SPAX5 patient cells. Targeted profiling included a range of metabolites essential to cellular energy pathways, such as intermediates of the TCA cycle, energy-related molecules (AMP, ADP, ATP), acetyl- and succinyl-CoA, as well as key compounds in glycolysis and amino acid metabolism. Despite this comprehensive approach, no statistically significant differences were observed in the metabolite levels when comparing the patient cells to healthy controls (*p* > 0.05, see Figure 3). These findings suggest that the metabolic disruptions inferred from proteomic data may not be directly reflected at the metabolite level or could involve complex regulatory mechanisms that are not immediately apparent through standard metabolite quantification.

### 3.4 Lipidomic profiling reveals significant alterations in lipid metabolism in SPAX5 lymphoblastoid cells

Lipidomic analysis of lymphoblastoid cells derived from a SPAX5 patient revealed significant alterations in lipid profiles compared to control cells: sphingomyelin, phosphatidylethanolamine, and phosphatidylcholine levels were significantly decreased in patient derived cells compared to controls (all *p* < 0.001). In contrast, triacylglycerol and lysophosphatidylcholine levels were significantly elevated in cells derived from the SPAX5 patient compared to controls (both *p* < 0.001). Additionally, cholesteryl ester levels were higher in patient-derived cells compared to controls (*p* < 0.001).

**FIGURE 4:**
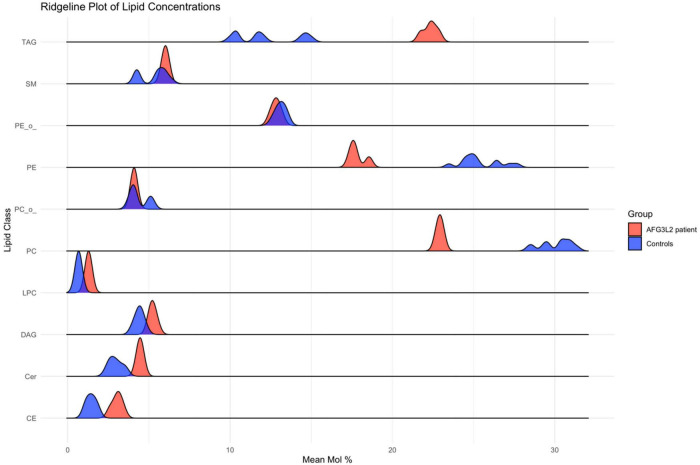
Results of lipid profiling on *AFG3L2*-mutant immortalized lymphoblastoid cells. Ridgeline plot depicting the distribution of lipid concentrations (Mean Mol %) across various lipid classes in AFG3L2 patients (red) and controls (blue). The *x*-axis represents the Mean Mol % of lipids, while the *y*-axis categorizes the lipid classes. This plot illustrates the density of lipid concentrations, enabling a comparative analysis of lipid profiles between AFG3L2 patients and controls. The plot demonstrates distinct distribution patterns, highlighting variations in lipid concentrations.

Other lipids, such as diacylglycerol and ceramide, did not demonstrate statistically significant differences between patient and control samples. These findings highlight alterations in lipid metabolism in SPAX5 patient derived lymphoblastoid cells, reflecting broader disruptions in overall cellular biochemical homeostasis (Figure 4).

## 4 Discussion

### 4.1 Pathogenic role of mitochondrial protease mutations in neurodegeneration

Mutations in mitochondrial proteases have been implicated in a number of human neurodegenerative diseases (Martinelli and Rugarli, 2010). AFG3L2 is a good example when a mitochondrial protease result in both dominant and recessive neurodegenerative diseases leading to ataxia and spastic paraparesis (SCA28 and SPAX5). Despite this, the precise understanding of the molecular etiology of the associated pathomechanisms still remains elusive. Moreover, the availability of suitable human *in vitro* models, crucial for elucidating disease mechanisms in SCA28 and/or SPAX5 and identifying minimally invasive cellular biomarkers, is still limited. To advance the understanding of neurodegeneration linked to impaired AFG3L2 function, we here investigated immortalized lymphoblastoid cells from a pediatric SPAX5 patient with compound heterozygous *AFG3L2* variants. A multi-level omics approach was applied to provide unbiased insights into the complex pathomechanism underlying SPAX5.

### 4.2 Clinical, imaging, and proteomic findings suggest rapid neurodegeneration accompanied by protein dysregulation

Neurodegeneration including optic nerve atrophy, spastic ataxia, and progressive cerebral atrophy is a clinical hallmark in patients suffering from pathogenic *AFG3L2* variants (Caporali et al., 2020; Jin et al., 2023; Pareek and Pallanck, 2020). On the imaging level, these clinical hallmarks are accompanied by brain volume loss and hydrocephalus. In line with this our patient‘s cranial MRI scans showing a dramatic and rapid progression of global and particularly cerebellar atrophy. Our proteomic findings show that the identified variants impact on AFG3L2 abundance and underline that the pathophysiological consequences upon loss of functional *AFG3L2* are not isolated to single cellular components but rather extend across a network of interrelated processes. Abnormally expressed proteins may impact different aspects of the neurodegenerative phenotype observed in our patient by perturbing diverse cellular processes reflecting areas critical for defects of *AFG3L2*. Screening of our proteomic data for proteins known to play crucial roles in neuron function, plasticity and neurodegeneration revealed a dysregulation of key proteins such as KCTD12 (Potassium Channel Tetramerization Domain Containing 12) and VRK2 (Vaccinia Related Kinase 2). KCTD12, an auxiliary subunit in GABAB receptor signaling, is implicated in stress response and psychiatric disorders, with stress increasing its expression in the hippocampus, affecting neuronal excitability and stress vulnerability (Deng et al., 2021). Similarly, VRK2, a kinase involved in cell cycle regulation, is crucial for maintaining neuronal health. Its dysregulation disrupts key pathways essential for neuronal function and survival, contributing to neurodegenerative diseases. VRK2 is known to affect the stability and activity of proteins such as JIP1 (c-Jun N-terminal kinase (JNK)-interacting protein 1) and dysbindin, which are vital for neuronal processes (Li and Yue, 2018).

### 4.3 Iron-sulfur cluster deficiency linked to immune dysregulation in SPAX5 lymphoblastoid cells

The downregulation of NFU1 (iron-sulfur cluster scaffold) observed in our proteomic data aligns with its critical role as an iron-sulfur cluster protein essential for mitochondrial function. NFU1 deficiency, as reported in cases of spastic paraparesis, exacerbates mitochondrial vulnerability and can lead to acute worsening during infections, which connects to our findings of heightened immune-related pathway activation (Tonduti et al., 2015; Uzunhan et al., 2020). This suggests that the immune dysregulation observed in our SPAX5 patient cells could stem, in part, from compromised NFU1 function, further destabilizing mitochondrial integrity under immune stress. The link between NFU1 and immune-triggered symptom escalation is particularly relevant given the immune pathway upregulations we detected, indicating an increased sensitivity of SPAX5 cells to inflammatory stress.

### 4.4 Calcium homeostasis disruption exacerbates mitochondrial instability

PRKCB dysregulation may contribute to the disrupted calcium homeostasis observed upon loss of functional *AFG3L2*. In this context, it is important to note that the MCU, which is affected in lymphoblastoid cells of our SPAX5 patient regulates calcium influx into mitochondria, a process crucial for cellular functions such as energy production, signal transduction, and apoptosis (De Stefani et al., 2015). The MCU complex includes multiple regulatory components–EMRE (essential MCU regulator), MICU1, MICU2, and, in some contexts, MICU3 (mitochondrial calcium uptake 1,2 and 3)–that act as gatekeepers, adjusting MCU activity based on calcium levels (Xing et al., 2019). AFG3L2 plays a key role in maintaining this balance by degrading excess EMRE, preventing its accumulation and ensuring proper MCU function. This regulation helps maintain cellular calcium equilibrium and mitochondrial integrity [(Tsai et al., 2017). Evidence suggests that PRKCB influences the dynamic interplay of intracellular calcium pools, thereby impacting mitochondrial calcium influx based on cellular demands (Pinton et al., 2004). Systematic studies on calcium homeostasis would be needed to further elucidate the impact of loss of functional AFG3L2 on abundance of this second messenger.

### 4.5 Protein dysregulations suggest cytoskeletal and vesicle transport dysfunction in SPAX5 lymphoblastoid cells

Our proteomic analysis further highlights key disruptions in cytoskeletal function and vesicle trafficking, specifically implicating MARCKSL1 (Myristoylated Alanine-Rich C Kinase Substrate-Like Protein 1) and Myosin-6, which are critical for actin filament regulation and vesicle transport, respectively (Brudvig and Weimer, 2015; Buss et al., 2001). Dysregulation of these proteins may hint toward a direct link between mitochondrial dysfunction, cytoskeletal instability, and impaired vesicular trafficking, potentially contributing to SPAX5 pathogenesis. Other proteins involved in cytoskeletal dynamics, such as WIPF1 (WASP-Interacting Protein Family Member 1) and TUBB6 (Tubulin Beta-6 Chain), were also dysregulated, further supporting a cascade of molecular events affecting cell structure and transport processes (Ferrer et al., 2001; Maurin et al., 2021; Moreau et al., 2000; Vemu et al., 2017). These findings underscore the critical interaction between mitochondrial dysfunction and cytoskeletal integrity, which may drive the disease mechanism in SPAX5. However, further imaging studies focusing on cytoskeletal integrity and vesicle trafficking are needed to pinpoint the exact impact of altered cytoskeleton and vesicle transport in SPAX5.

### 4.6 Affection of energy metabolism relevant proteins in SPAX5

*In silico* analysis points to metabolic stress, suggesting disruptions in various cellular metabolic activities. However, despite targeted profiling of a broad array of metabolites–including intermediates of the TCA cycle, molecules involved in oxidative phosphorylation, glycolysis, and amino acid metabolism–no statistically significant differences were observed. The lack of statistically significant differences in the metabolic data could be due to variability within the control group compared to the single patient sample, as well as potential metabolic differences between lymphoblastoid cells and neurons. Nevertheless, altered mitochondrial proteins in our patient’s cells are known to impact on energy metabolism and respiration, suggesting a potential link between mitochondrial dysfunction and energy management in SPAX5. The dysregulation of COX11 (Cytochrome c oxidase assembly protein COX11), essential for cytochrome c oxidase in the electron transport chain, may impair ATP production, reflecting broader challenges in maintaining cellular energy demands (Carr et al., 2002). The observed proteomic changes support a model of mitochondrial dysfunction impacting cellular energy management, despite the absence of direct metabolic alterations in the profiling data. This in turn suggests that regulatory mechanisms may buffer metabolic shifts at the level of metabolite concentrations, potentially allowing cells to maintain stable energy-related metabolite levels even in the presence of mitochondrial protein dysregulation. Further investigation is warranted to explore these regulatory processes and their potential compensatory roles in maintaining cellular homeostasis in SPAX5.

### 4.7 Lipid metabolism shifts highlight membrane instability and inflammatory state

The observed lipidomic alterations highlight disruptions in lipid metabolism associated with *AFG3L2* mutations. Reductions in sphingomyelin, phosphatidylethanolamine, and phosphatidylcholine suggest compromised membrane integrity, aligning with mitochondrial dysfunction and oxidative stress known in SPAX5, as these lipids are essential for maintaining mitochondrial membrane integrity and limiting oxidative damage (Hannun and Obeid, 2008; Vance, 2015). Increased levels of triacylglycerol, lysophosphatidylcholine, and cholesteryl ester in patient cells may indicate compensatory mechanisms against metabolic stress, with triacylglycerol storage possibly serving to mitigate lipotoxicity (Listenberger et al., 2003; Walther and Farese, 2009).

The rise in lysophosphatidylcholine, a lipid involved in inflammatory responses, points to a potential inflammatory state in patient cells, consistent with previously reported neuroinflammatory aspects of *AFG3L2* (Knuplez and Marsche, 2020; Liu et al., 2020); however, MRI results showed no signs of neuroinflammation, although clinical follow-ups reveal a higher infection susceptibility. Proteomic findings also reveal dysregulation of proteins like ISG15 (Interferon-stimulated gene 15) and OASL (2′-5′-Oligoadenylate Synthetase Like), which are linked to neuroinflammation (Clarkson et al., 2022; Zhang et al., 2023). Additionally, cholesteryl ester increases may reflect disruptions in cholesterol homeostasis, essential for membrane fluidity and cellular stability (Cooper, 1978). Elevated lysophosphatidylcholine may further impact membrane curvature and vesicle formation, potentially affecting protein trafficking and synaptic function. Further multi-omics studies, particularly on isolated extracellular vesicles, are needed to clarify the role of altered vesicle trafficking and homeostasis in SPAX5 pathophysiology.

### 4.8 Limitations

In light of these findings, we recognize that while our study offers significant insights into the molecular consequences of *AFG3L2* mutations, it also presents opportunities for further exploration. While lymphoblastoid cell lines provide a valuable model system, they may not fully capture the complexity of neurodegenerative processes in SPAX5, which would require neuronal or *in vivo* models for deeper investigation. Additionally, our analysis focuses on a single time point, which may overlook dynamic changes in cellular pathways over time for which studies on animal models would be best suitable. Expanding the sample size and incorporating longitudinal studies could further enhance the robustness of our findings.

Moreover, the scope of our study is limited by focusing on a single patient with specific *AFG3L2* variants. Future research would benefit from examining multiple patients with a range of *AFG3L2* mutations, as analyzing diverse variants and associated phenotypes could reveal mutation-specific molecular mechanisms and offer insights into the varied clinical presentations observed in SPAX5.

## 5 Conclusion

Our comprehensive multi-omics based investigation focusing on cellular consequences upon loss of functional *AFG3L2* not only allowed to demonstrate an impact of the genetic variants on the corresponding protein but also enabled to elucidate a pathophysiology that extends beyond the boundaries of mitochondrial proteostasis, implicating a wide array of cellular processes. While mitochondrial dysfunction is a significant component, our findings reveal substantial cellular disturbances on a multi-layer level, which may contribute to neurodegeneration. This is not only based on altered proteins playing important roles in neuronal function but also on affected mechanisms such as abundance of proteins crucial for mitochondrial calcium homeostasis and cytoskeletal integrity in addition to altered metabolic and lipid homeostasis. By unveiling these biochemical networks, we provided deeper insights into the cascade of molecular events leading to cellular dysfunction and consequently disease manifestation of SPAX5. We propose that our combined omics data not only provide novel insights into the complex pathobiochemistry underlying SPAX5 on a molecular multi-layer level but also introduce a biochemical fingerprint that can be monitored in patient-derived white blood cells as minimally invasive biomarkers with clinical relevance. However, although these findings enhance our understanding of the disease’s underlying mechanisms, it is evident that further research is needed to fully unravel these complex interactions of pathophysiological cascades in terms of molecular dissection of primary and secondary events. Of note, such knowledge is crucial in identifying potential therapeutic targets aimed at restoring both mitochondrial and overall cellular health. Along this line, our data suggest that SPAX5 patient-derived blood cells may serve as an *in vitro* system for pre-clinical interventions, where restoration of biochemical homeostasis could serve as a reliable outcome measure.

## Data Availability

The original contributions presented in the study are included in the article/supplementary material, further inquiries can be directed to the corresponding authors.

## Supplementary material

Supplementary Figure 1 GO term-based analysis of DAPs. GO term-based *in silico* study of DAPs was carried out for increased and decreased proteins separately to decipher molecular processes and subcellular structures primarily affected by protein dysregulations. DAPs with increased abundance impact on phagocytosis, protein folding, SMAD protein complex assembly, supramolecular fiber organization and actin filament-based movement, while downregulated DAPs are associated with pore formation, membrane raft assembly, retinol metabolic process, and Golgi lumen function.

Supplementary Table 1 Proteomic analysis of *AFG3L2*-mutant immortalized lymphoblastoid cells.
