# Supplementary figures and images for: Multi-omics-based phenotyping of AFG3L2-mutant lymphoblasts determines key factors of a pathophysiological interplay between mitochondrial vulnerability and neurodegeneration in spastic ataxia type 5
Source: Front Mol Neurosci. 2025 Feb 20;18:1548255. doi: 10.3389/fnmol.2025.1548255 (PMC11882581; doi:10.3389/fnmol.2025.1548255)

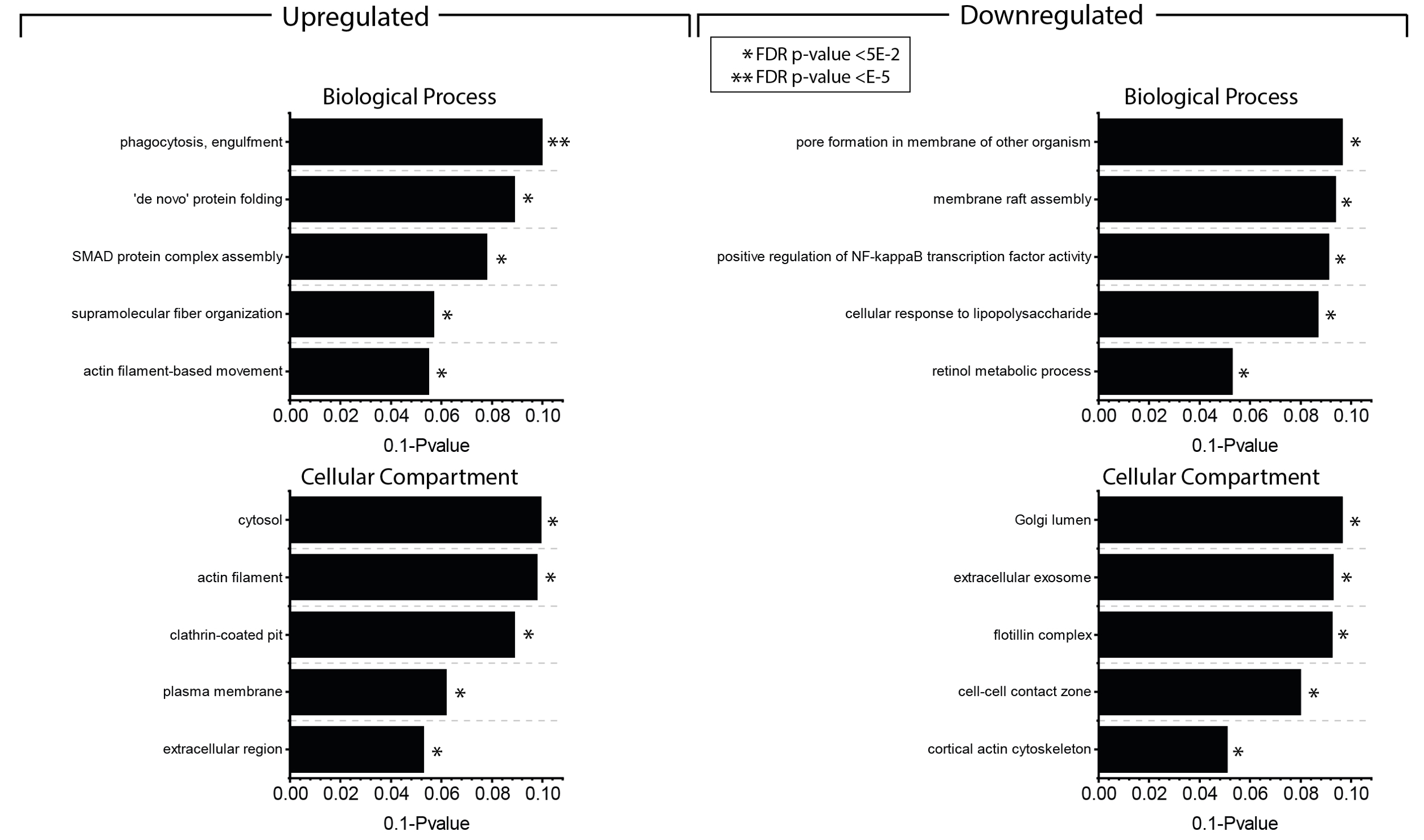

Supplement: Supplementary Figure 1 — GO term-based analysis of DAPs. GO term-based in silico study of DAPs was carried out for increased and decreased proteins separately to decipher molecular processes and subcellular structures primarily affected by protein dysregulations. DAPs with increased abundance impact on phagocytosis, protein folding, SMAD protein complex assembly, supramolecular fiber organization and actin filament-based movement, while downregulated DAPs are associated with pore formation, membrane raft assembly, retinol metabolic process, and Golgi lumen function. [file Image_1.jpeg]
