# Supplementary material for: Multi-omics-based phenotyping of AFG3L2-mutant lymphoblasts determines key factors of a pathophysiological interplay between mitochondrial vulnerability and neurodegeneration in spastic ataxia type 5
Source: Front Mol Neurosci. 2025 Feb 20;18:1548255. doi: 10.3389/fnmol.2025.1548255 (PMC11882581; doi:10.3389/fnmol.2025.1548255)
Supplement: Supplementary Table 1 — Proteomic analysis of AFG3L2-mutant immortalized lymphoblastoid cells. [file Table_1.docx]

| **Upregulated Proteins** | | |  |  |
| --- | --- | --- | --- | --- |
| **PG.ProteinAccessions** | **PG.Genes** | **PG.ProteinNames** | **Patient/Control** | **P value** |
| P01857 | IGHG1 | IGHG1_HUMAN | 61,71 | 0,00 |
| A0A0C4DH41 | IGHV4-61 | HV461_HUMAN | 31,44 | 0,00 |
| P01860 | IGHG3 | IGHG3_HUMAN | 14,64 | 0,00 |
| P06454 | PTMA | PTMA_HUMAN | 6,40 | 0,00 |
| P09914 | IFIT1 | IFIT1_HUMAN | 5,10 | 0,00 |
| P49006 | MARCKSL1 | MRP_HUMAN | 4,36 | 0,00 |
| Q9UM54 | MYO6 | MYO6_HUMAN | 3,75 | 0,00 |
| P02794 | FTH1 | FRIH_HUMAN | 3,72 | 0,00 |
| P21926 | CD9 | CD9_HUMAN | 3,65 | 0,00 |
| P05161 | ISG15 | ISG15_HUMAN | 3,58 | 0,00 |
| Q15646 | OASL | OASL_HUMAN | 3,24 | 0,00 |
| O14879 | IFIT3 | IFIT3_HUMAN | 3,24 | 0,00 |
| P09913 | IFIT2 | IFIT2_HUMAN | 3,23 | 0,00 |
| Q7KZN9 | COX15 | COX15_HUMAN | 3,14 | 0,01 |
| Q5EBM0 | CMPK2 | CMPK2_HUMAN | 3,06 | 0,00 |
| P48595 | SERPINB10 | SPB10_HUMAN | 3,06 | 0,00 |
| Q8IY21 | DDX60 | DDX60_HUMAN | 2,89 | 0,01 |
| O15162 | PLSCR1 | PLS1_HUMAN | 2,82 | 0,00 |
| P24557 | TBXAS1 | THAS_HUMAN | 2,67 | 0,00 |
| B9A064 | IGLL5 | IGLL5_HUMAN | 2,64 | 0,05 |
| P62942 | FKBP1A | FKB1A_HUMAN | 2,62 | 0,05 |
| Q9UMS0 | NFU1 | NFU1_HUMAN | 2,59 | 0,02 |
| O43516 | WIPF1 | WIPF1_HUMAN | 2,50 | 0,01 |
| Q27J81 | INF2 | INF2_HUMAN | 2,43 | 0,00 |
| Q9Y6K5 | OAS3 | OAS3_HUMAN | 2,36 | 0,00 |
| Q08AF3 | SLFN5 | SLFN5_HUMAN | 2,35 | 0,00 |
| Q9BS40 | LXN | LXN_HUMAN | 2,32 | 0,01 |
| Q9NZZ3 | CHMP5 | CHMP5_HUMAN | 2,30 | 0,00 |
| Q96CX2 | KCTD12 | KCD12_HUMAN | 2,28 | 0,02 |
| P05114 | HMGN1 | HMGN1_HUMAN | 2,27 | 0,04 |
| P49407 | ARRB1 | ARRB1_HUMAN | 2,23 | 0,01 |
| P28907 | CD38 | CD38_HUMAN | 2,23 | 0,05 |
| Q9Y6N1 | COX11 | COX11_HUMAN | 2,22 | 0,04 |
| Q14914 | PTGR1 | PTGR1_HUMAN | 2,21 | 0,00 |
| P07311 | ACYP1 | ACYP1_HUMAN | 2,19 | 0,00 |
| P50440 | GATM | GATM_HUMAN | 2,18 | 0,00 |
| P36959 | GMPR | GMPR1_HUMAN | 2,18 | 0,01 |
| Q13325 | IFIT5 | IFIT5_HUMAN | 2,17 | 0,01 |
| Q96GG9 | DCUN1D1 | DCNL1_HUMAN | 2,13 | 0,01 |
| Q86Y07 | VRK2 | VRK2_HUMAN | 2,10 | 0,01 |
| Q14247 | CTTN | SRC8_HUMAN | 2,07 | 0,00 |
| Q9UDT6 | CLIP2 | CLIP2_HUMAN | 2,07 | 0,04 |
| Q9BUF5 | TUBB6 | TBB6_HUMAN | 2,06 | 0,02 |
| Q9H9A5 | CNOT10 | CNO10_HUMAN | 2,06 | 0,02 |
| P21980 | TGM2 | TGM2_HUMAN | 2,05 | 0,00 |
| Q16850 | CYP51A1 | CP51A_HUMAN | 2,00 | 0,00 |
| P17655 | CAPN2 | CAN2_HUMAN | 2,00 | 0,00 |

| **Downregulated Proteins** | | |  |  |
| --- | --- | --- | --- | --- |
| **PG.ProteinAccessions** | **PG.Genes** | **PG.ProteinNames** | **Patient/Control** | **P value** |
| P05771 | PRKCB | KPCB_HUMAN | 0,49 | 0,01 |
| Q9HD23 | MRS2 | MRS2_HUMAN | 0,48 | 0,04 |
| Q9NSI8 | SAMSN1 | SAMN1_HUMAN | 0,48 | 0,05 |
| Q8TCD5 | NT5C | NT5C_HUMAN | 0,47 | 0,02 |
| Q9BTZ2 | DHRS4 | DHRS4_HUMAN | 0,45 | 0,00 |
| Q9BX59 | TAPBPL | TPSNR_HUMAN | 0,42 | 0,05 |
| P59665;P59666 | DEFA1;DEFA3 | DEF1_HUMAN | 0,42 | 0,03 |
| Q9H939 | PSTPIP2 | PPIP2_HUMAN | 0,40 | 0,05 |
| P06340 | HLA-DOA | DOA_HUMAN | 0,39 | 0,04 |
| P49441 | INPP1 | INPP_HUMAN | 0,37 | 0,01 |
| Q6ZUJ8 | PIK3AP1 | BCAP_HUMAN | 0,34 | 0,00 |
| Q9NR19 | ACSS2 | ACSA_HUMAN | 0,30 | 0,04 |
| Q8NBN7 | RDH13 | RDH13_HUMAN | 0,28 | 0,00 |
| Q6ZSS7 | MFSD6 | MFSD6_HUMAN | 0,27 | 0,02 |
| Q14254 | FLOT2 | FLOT2_HUMAN | 0,22 | 0,01 |
| O75955 | FLOT1 | FLOT1_HUMAN | 0,20 | 0,00 |
